# Rats Genetically Selected for High Aerobic Exercise Capacity Have Elevated Plasma Bilirubin by Upregulation of Hepatic Biliverdin Reductase-A (BVRA) and Suppression of UGT1A1

**DOI:** 10.3390/antiox9090889

**Published:** 2020-09-19

**Authors:** Terry D. Hinds, Justin F. Creeden, Darren M. Gordon, Adam C. Spegele, Steven L. Britton, Lauren G. Koch, David E. Stec

**Affiliations:** 1 Department of Pharmacology and Nutritional Sciences, University of Kentucky College of Medicine, Lexington, KY 40508, USA; 2 Department of Neurosciences, University of Toledo College of Medicine, Toledo, OH 43614, USA; Justin.Creeden@rockets.utoledo.edu (J.F.C.); Darren.Gordon@rockets.utoledo.edu (D.M.G.); 3 Department of Physiology and Pharmacology, University of Toledo College of Medicine, Toledo, OH 43614, USA; Adam.Spegele@rockets.utoledo.edu (A.C.S.); Lauren.Koch2@utoledo.edu (L.G.K.); 4 Department of Anesthesiology, Department of Molecular and Integrative Physiology, University of Michigan, Ann Arbor, MI 48109, USA; brittons@umich.edu; 5 Center for Excellence in Cardiovascular-Renal Research, Department of Physiology & Biophysics, University of Mississippi Medical Center, 2500 North State St, Jackson, MS 392161, USA

**Keywords:** bilirubin, BVRA, exercise, obesity, PPARalpha, glycogen, mitochondria, heme oxygenase, HO-1, HO-2

## Abstract

Exercise in humans and animals increases plasma bilirubin levels, but the mechanism by which this occurs is unknown. In the present study, we utilized rats genetically selected for high capacity running (HCR) and low capacity running (LCR) to determine pathways in the liver that aerobic exercise modifies to control plasma bilirubin. The HCR rats, compared to the LCR, exhibited significantly higher levels of plasma bilirubin and the hepatic enzyme that produces it, biliverdin reductase-A (BVRA). The HCR also had reduced expression of the glucuronyl hepatic enzyme UGT1A1, which lowers plasma bilirubin. Recently, bilirubin has been shown to activate the peroxisome proliferator-activated receptor-α (PPARα), a ligand-induced transcription factor, and the higher bilirubin HCR rats had significantly increased PPARα-target genes *Fgf21*, *Abcd3*, and *Gys2*. These are known to promote liver function and glycogen storage, which we found by Periodic acid–Schiff (PAS) staining that hepatic glycogen content was higher in the HCR versus the LCR. Our results demonstrate that exercise stimulates pathways that raise plasma bilirubin through alterations in hepatic enzymes involved in bilirubin synthesis and metabolism, improving liver function, and glycogen content. These mechanisms may explain the beneficial effects of exercise on plasma bilirubin levels and health in humans.

## 1. Introduction

Genetics or a sedentary lifestyle may lead to obesity and other deleterious comorbidities that can reduce life expectancy. Exercise and diet modifications are beneficial in reducing body weight and adverse outcomes. Recent investigations have shown that bilirubin, which was typically considered a toxic bile substance, has beneficial actions on regulating body weight [1,2]. Indeed, there has been a correlation showing that plasma bilirubin levels are lower in obese humans [3,4,5]. The enzyme that produces bilirubin, BVRA [6,7], is also reduced in obese humans compared to lean matched controls [8]. Studies indicate that bilirubin levels might increase with exercise [9,10] and fasting [11,12,13,14], implying a possible role for metabolic regulation.

There are multiple ways of possibly regulating plasma bilirubin, which is a product of the heme oxygenase (HO) pathway [15]. Heme from lysed red blood cells is broken down to biliverdin by HO (HO-1 or HO-2), and this is further catabolized by biliverdin reductase (BVR) to unconjugated bilirubin [16]. This version of bilirubin is insoluble and requires glucuronidation by the UGT1A1 enzyme for solubility [17], and then it is deposited in the bile, and eventually, the intestine, ultimately reducing plasma levels [16]. Hence, regulation of HO, BVR, or UGT1A1 can all serve as mechanisms to regulate plasma bilirubin levels and possibly body weight. However, how these enzymes function during exercise to modify bilirubin levels is unknown.

We have shown that bilirubin has a hormonal function by binding directly to the fat-burning nuclear receptor PPARα inducing transcriptional activity and gene regulation [18,19,20]. PPARα is induced by fasting [21], which is also when bilirubin is the highest [11,12,13,14]. There may be a bilirubin-induced endocrine function during fasting, which has been shown for PPARα-mediated induction of fibroblast growth factor 21 (FGF21) [22]. We have previously demonstrated that bilirubin-PPARα increased FGF21 levels, activating mitochondrial function and β-oxidation for utilization of fats for energy reducing body weight [18,19,23,24,25]. The function of bilirubin-PPARα in exercise has not been explored.

While most studies have focused on HO-1 expression and bilirubin as an end product, in this study, we analyzed each enzyme in the HO pathway in rats genetically selected for high and low aerobic exercise capacity. We found that the BVRA is enhanced in the high capacity running (HCR) compared to the low capacity running (LCR) rats. Also, we found that UGT1A1 expression was reduced, and together these caused higher plasma bilirubin levels and PPARα-induced hepatic β-oxidation pathway. Together, these posit that bilirubin may function to enhance exercise and aerobic capacity, and this may help reduce body fat. Increasing serum bilirubin by targeting the HO-BVR-UGT1A1 pathway, which are enzymes that regulate its production or turnover, might be a useful therapeutic approach for exercise performance or obesity.

## 2. Materials and Methods

### 2.1. Animals

Studies were performed on male rats 59–63 weeks of age from generation 41 of HCR/LCR rat model, which was developed by two-way selective breeding in genetically heterogeneous rats for intrinsic exercise capacity as previously described [26,27,28,29]. In short, rats were phenotyped at 11 weeks of age using a speed-ramped maximal treadmill running test, and we devised to pattern clinical stress tests. The experimental procedures and protocols of this study were approved by the Institutional Animal Care and Use Committee of the University of Toledo Health Science Center and conformed to the National Institutes of Health Guide for the Care and Use of Laboratory Animals. Rats were maintained on standard laboratory diet (Laboratory Rodent Diet 5001; LabDiet, Richmond, IN, USA) with free access to water throughout the study. The running protocol consisted of two days of re-education to treadmill walking for no more than 5 min, followed by a single test of maximal treadmill running distance carried on day 3. The treadmill (model Exer-4, Columbus Instruments, Columbus, OH, USA) was set at a constant grade of 15° and an initial speed of 10 m/min. Speed was progressively ramped 1 m/min every 2 min until the rat could no longer keep pace and continue running. Total run time and body weight were recorded. The distance run in meters (m) was calculated from treadmill speed (m/min). The next day, rats were fasted overnight prior to the terminal collection of blood and tissue samples in rats deeply anesthetized with isoflurane induction.

### 2.2. Liver Histology

To determine hepatic differences of the HCR and LCR rats, livers were mounted and frozen in Tissue-Tek O.C.T and sectioned at 10 µm. Hematoxylin and Eosin (H&E) and Periodic acid–Schiff (PAS) staining were performed as previously described [23,24,30]. Frozen sections were air-dried and fixed in 10% neutral buffered formalin. Sections were briefly rinsed in tap water followed by 60% isopropanol and stained for 15 min in the Oil Red O solution. Sections were further rinsed in 60% isopropanol and nuclei stained with hematoxylin followed by aqueous mounting and coverslipping. Slides were imaged at 20× magnification using a color video camera attached to an Olympus VS120 slide scanning microscope (Olympus Corporation, Center Valley, PA, USA). Images were analyzed using the Olympus OlyVIA software. Image J (NIH) was used to quantitate the glycogen content. Data are presented as the + SEM for each group.

### 2.3. Quantification of Small Molecules in Plasma

Were measured as we previously described [30]. In brief, following an overnight fast, blood samples were obtained via the abdominal aorta under isoflurane anesthesia for plasma lipids and metabolites. Nuclear magnetic resonance (NMR) spectroscopy experiments were acquired using a 14.0 T Bruker magnet equipped with a Bruker AV-III console operating at 600.13 MHz. (Bruker Scientific LLC, Billerica, MA, USA). All spectra were acquired in 3 mm NMR tubes using a Bruker 5 mm QCI cryogenically cooled NMR probe. Plasma samples were prepared and analyzed according to the Bruker In-Vitro Diagnostics research (IVDr) protocol. Sample preparation consisted of combining 50 μL of plasma with 150 μL of the buffer supplied by Bruker Biospin specifically for the IVDr protocol. For 1D ^1^H NMR, data was acquired using the 1D-NOE experiment, which filters NMR signals associated with broad line widths arising from proteins that might be present in plasma samples that adversely affect spectral quality. Experiment conditions included: Sample temperature of 310K, 96k data points, 20 ppm sweep width, a recycle delay of 4 s, a mixing time of 150 ms, and 32 scans. The analysis was performed using a regression analysis of the NMR data, which is done automatically as part of the IVDr platform, as previously described [31].

### 2.4. Quantitative Real-Time PCR Analysis

Total RNA was harvested from HCR and LCR rats by lysing livers using a Qiagen Tissue Lyser LT (Qiagen Inc, Germantown, MD, USA) and then extraction by 5-Prime PerfectPure RNA Tissue Kit (Fisher Scientific Company, LLC). Total RNA was read on a NanoDrop 2000 spectrophotometer (Thermo Fisher Scientific, Wilmington, DE, USA), and cDNA was synthesized using High Capacity cDNA Reverse Transcription Kit (Applied Biosystems). PCR amplification of the cDNA was performed by quantitative real-time PCR using TrueAmp SYBR Green qPCR SuperMix (Alkali Scientific, Fort Lauderdale, FL, USA) for gene-specific primers as previously described [18,23,24,32]. The thermocycling protocol consisted of 5 min at 95 °C, 40 cycles of 15 s at 95 °C, and 30 s at 60 °C, finished with a melting curve ranging from 60–95 °C to allow distinction of specific products. Normalization was performed in separate reactions with primers to GAPDH.

### 2.5. Bilirubin Measurements

Total bilirubin was measured using a Vet Axcel serum chemistry analyzer (AlfaWassermann, West Caldwell, NJ, USA) from 30 μL of plasma. Samples were measured in duplicate with standards supplied by the manufacturer, as previously described [20]. Data are presented as mg/dL.

### 2.6. Statistics

All data are presented as mean + S.E.M. Differences between treatment groups were determined using the student t-test. A *p* < 0.05 was considered to be significant. All analyses were performed with GraphPad Prism 8 software (GraphPad Software, Inc., San Diego, CA, USA).

## 3. Results

**Figure 1:**
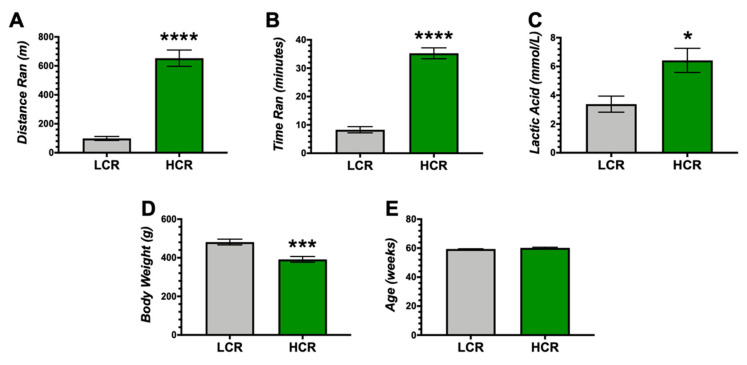
Phenotypic differences between low capacity running (LCR) and high capacity running (HCR) rats. (**A**) Distance ran to exhaustion. (**B**) Time ran to exhaustion. (**C**) Blood lactic acid levels. (**D**) Body Weight. (**E**) Age of rats. * *p* < 0.05 as compared to LCR rats. *** *p* < 0.01 as compared to LCR rats. **** *p* < 0.001 as compared to LCR rats. n = 9/group.

The HCR and LCR total running distance were measured, and as expected [33] the HCR rats had significantly (*p* < 0.0001) ran further and had higher time to exhaustion during running (Figure 1A,B).

Exercise capacity increases lactic acid in the blood [34,35], and this was also found to be significantly (*p* < 0.05) higher in the HCR rats (Figure 1C). The higher aerobic exercise HCR rats had reduced body weights compared to the LCR (Figure 1D). Age of the rats did not affect these outcomes, as there was no difference in the age of the rats (Figure 1E).

**Figure 2:**
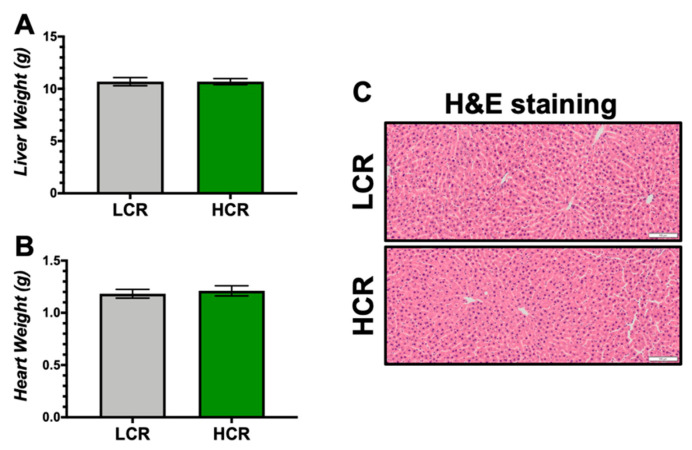
Organ weights and hepatic Hematoxylin and Eosin (H&E) staining in LCR and HCR rats. (**A**) Liver weight. (**B**) Heart weight. (**C**) Representative hepatic H&E staining. Scale bar = 100 μm. n = 9/group.

There was also no difference in liver or heart weights (Figure 2A,B). Histological H&E staining in the HCR and LCR rats did not show any presence of lipid droplets (steatosis), fibrosi s, or inflammatory foci. Overall, there were no noticeable differences in the hepatic visualization between the HCR and LCR rats.

**Figure 3:**
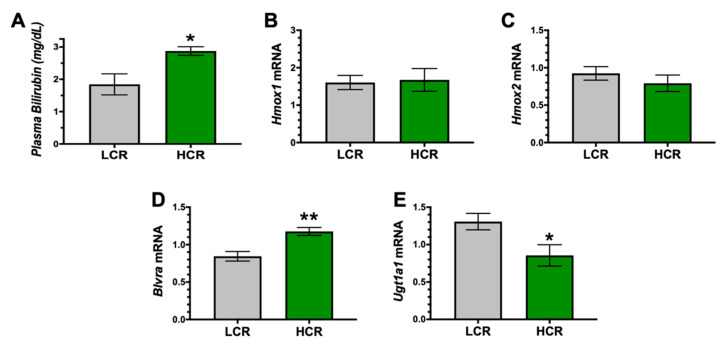
Plasma bilirubin and hepatic heme oxygenase pathway in LCR and HCR rats. (**A**) Plasma bilirubin levels. (**B**) Hepatic heme oxygense-1 (*Hmox1*) mRNA levels. (**C**) Hepatic heme oxygenase-2 (*Hmox2*) mRNA levels. (**D**) Hepatic biliverdin reductase-A (*Blvra*) mRNA levels. (**E**) Hepatic UDP Glucuronosyltransferase Family 1 Member A1 (*Ugt1a1*). * *p* < 0.05 as compared to LCR rats. ** *p* < 0.001 as compared to LCR rats. n = 9/group.

Bilirubin has been shown to increase with exercise capacity in humans [9,10]. To determine possible differential in hepatic function between the HCR and LCR rats, we measured plasma bilirubin levels, which is a surrogate marker of liver function. We found that plasma bilirubin levels were significantly (*p* < 0.05) higher in the HCR high aerobic running rats compared to the LCR (Figure 3A). We measured the enzyme expression of the HO pathway to determine if they were altered with high aerobic exercise capacity HCR rats. We found no differences between the HCR and LCR rats for the HO isoforms, *Hmox1* (HO-1), or *Hmox2* (HO-2) (Figure 3B,C). However, the enzyme that generates bilirubin, BVRA (*Blvra* gene), was significantly (*p* < 0.01) higher in the HCR rats. The enzyme that reduces plasma bilirubin levels, UGT1A1 (*Ugt1a1* gene), was significantly (*p* < 0.05) reduced in the HCR. These results show that higher aerobic exercise capacity enhanced BVRA and reduced UGT1A1 to elevate bilirubin levels.

**Figure 4:**
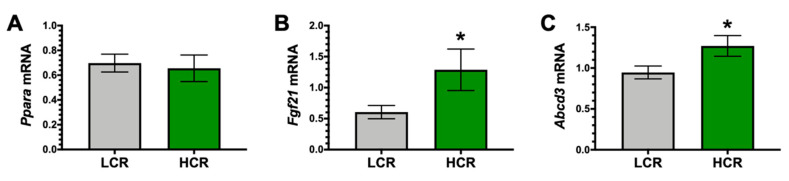
Hepatic peroxisome proliferator-activated receptor-α (PPARα) and target genes in LCR and HCR rats. (**A)** Hepatic PPARα (*Ppara*) mRNA levels. (**B**) Hepatic fibroblast growth factor-21 (*Fgf21*) mRNA levels. (**C**) Hepatic ATP Binding Cassette Subfamily D Member 3 (*Abcd3*) mRNA levels. * *p* < 0.05 as compared to LCR rats. n = 9/group.

Bilirubin has been shown to activate the transcriptional activity of the nuclear receptor PPARα for gene regulation [18,19,20]. We found no differences in the PPARα mRNA expression (*Ppara*) between the HCR and LCR (Figure 4A). However, we found that PPARα-target genes FGF21 (*Fgf21*) and *Abcd3* [30] were significantly (*p* < 0.05) higher in the HCR compared to the LCR rats (Figure 4B,C). Noland et al. reported that the HCR rats had higher fatty acid oxidation than the LCR [36], which agrees with our findings.

**Figure 5:**
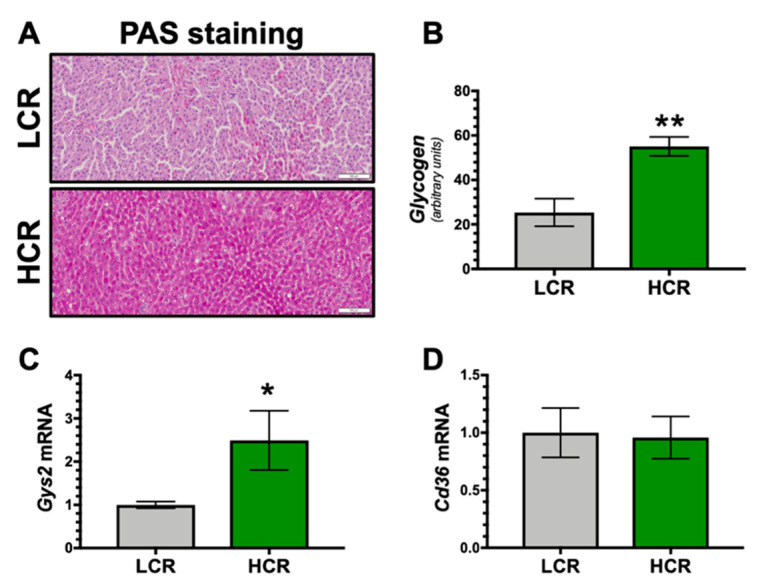
Hepatic glycogen and C36 levels in LCR and HCR rats. (**A**) Representative hepatic Periodic acid–Schiff (PAS) staining. (**B**) Quantification of hepatic glycogen staining. (**C**) Hepatic glycogen synthase-2 (*Gys2*) mRNA levels. (**D**) Hepatic CD36 (*Cd36*) mRNA levels. * *p* < 0.05 as compared to LCR rats. ** *p* < 0.001 as compared to LCR rats. Scale bar = 100 μm. n = 9/group.

We have shown that bilirubin-PPARα activates glycogen storage in the liver of obese mice [24]. Here, we observed that the HCR rats had significantly (p < 0.01) higher glycogen levels compared to the LCR (Figure 5A,B). The PPARα-target gene *Gys2* (glycogen synthase-2) [30,37], was significantly (*p* < 0.05) higher in the HCR rats, but not the glucose and fat scavenger *Cd36* (Figure 5C,D).

**Figure 6:**
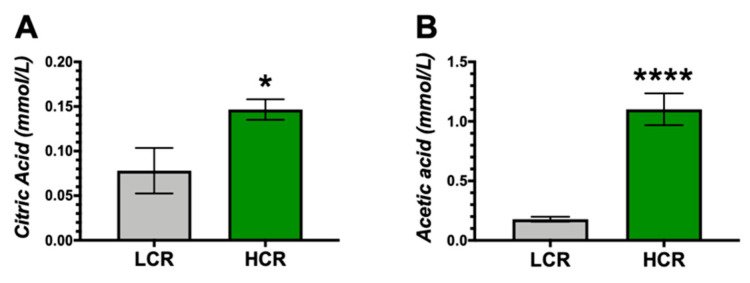
Plasma markers of mitochondrial activity in LCR and HCR rats. (**A**) Plasma levels of citric acid. (**B**) Plasma levels of acetic acid. * *p* < 0.05 as compared to LCR rats. **** *p* < 0.001 as compared to LCR rats. n = 9/group.

We have shown that bilirubin activation of PPARα induces mitochondrial function [20], and that mice with the loss of BVRA have reduced mitochondrial function [38]. Therefore, we measured citric acid and acetic acid in the HCR and LCR rats, two metabolites associated with mitochondrial activity [39,40,41]. The HCR rats had significantly increased levels of citric acid and acetic acid (Figure 6A,B).

**Figure 7:**
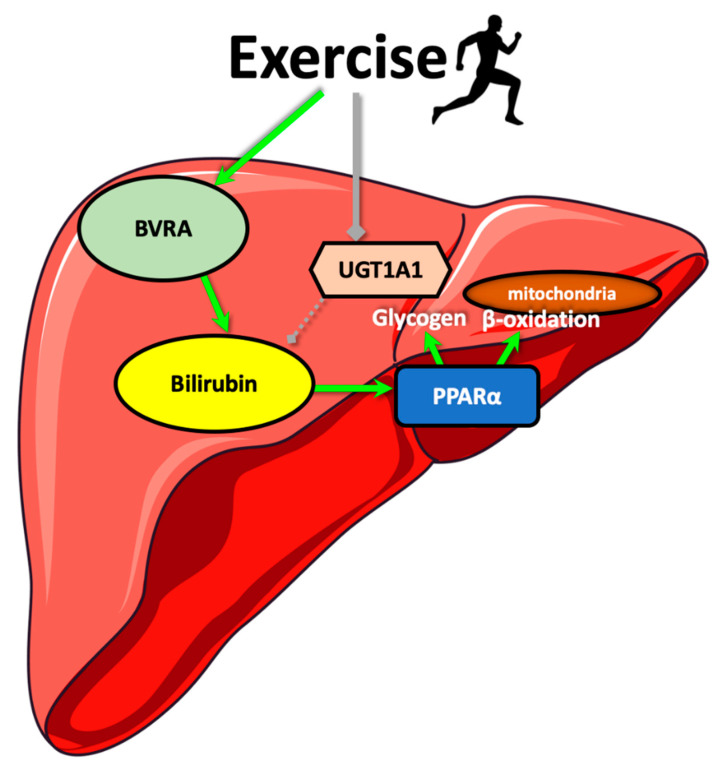
Schematic diagram of the proposed pathway by which exercise regulates plasma bilirubin levels. Exercise upregulates hepatic biliverdin reductase-A (BVRA) and suppresses UDP Glucuronosyltransferase Family 1 Member A1 (UGT1A1) resulting in increased plasma levels of bilirubin. Bilirubin then activates hepatic peroxisome proliferator-activated receptor-*α* (PPARα) and its target genes to enhance glycogen synthesis and mitochondrial β-oxidation.

These data indicate that HCR rats may have a higher running capacity by increasing hepatic BVRA, decreasing UGT1A1, elevating plasma bilirubin that activates PPARα for glycogen storage, and improvement of mitochondria function (Figure 7).

## 4. Discussion

Physical exercise provides multiple benefits to the body, and a sedentary lifestyle and weight gain are associated with an increased risk of obesity, cardiovascular, and metabolic diseases [42,43]. Previous studies have found that exercise increases serum bilirubin levels [9,10]. In the past, bilirubin has been negatively viewed as a toxic bile substance and a biomarker for liver dysfunction. This was mostly reflected from the severe hyperbilirubinemia observed in the extremely rare Crigler–Najjar syndrome with levels in the 400–700 μM range, which can cause brain damage in infants [44]. The past two decades of heme oxygenase and bilirubin research has shown that bilirubin has many health benefits [1,2,45]. For instance, humans with the UGT1A1*28 Gilbert’s polymorphism that causes reduced levels of the UGT1A1 enzyme and increased plasma bilirubin levels in the 18–50 μM range [46,47], have been shown to have decreased incidence of cardiovascular disease [48,49]. People exhibiting mildly elevated bilirubin levels (>12 μM) were shown to have significantly fewer metabolic disorders such as fatty liver disease, obesity, or type II diabetes [5,50,51,52,53,54,55,56]. These studies have opened a new perspective for bilirubin and that there are health benefits with mild hyperbilirubinemia.

The results of the present study showed that aerobic exercise increases hepatic BVRA expression while at the same time, suppressing UGT1A1. BVRA is the rate-limiting enzyme in the formation of bilirubin. UGT1A1 is the enzyme in the liver responsible for the conjugation of bilirubin so that it can be eliminated in the bile. Genetically deficient mice and animals treated with UGT1A1 antagonist or antisense oligonucleotides exhibit an increase in serum bilirubin levels according to the degree of UGT1A1 inhibition [23,57,58]. We have previously shown that mice with the human UGT1A1*28 Gilbert’s polymorphism and hyperbilirubinemia on a high-fat diet had significantly reduced hepatic fat accumulation and increased liver glycogen content compared to controls [23]. These indicate that mildly elevating plasma bilirubin levels have beneficial effects on liver pathology. While past studies have demonstrated the relationship between exercise and serum bilirubin levels, this is the first study to identify a potential mechanism by which exercise regulates serum bilirubin levels. HCR rats are a genetic model of high aerobic capacity similar to highly conditioned human athletes. Interestingly, a study of participants in a 1600 km ultramarathon run, which occurred over 16 days, found elevations in serum bilirubin levels at the end of the run [59]. This study did not measure the factors controlling the higher bilirubin levels. However, our results show that aerobic exercise induces hepatic BVRA and suppresses UGT1A1 to elevate plasma bilirubin.

Understanding the potential physiological benefit of increased bilirubin released during exercise is necessary to uncovering bilirubin’s diverse metabolic functions. One study showed in humans that increased aerobic exercise is correlated with higher levels of total bilirubin [9]. Another study demonstrated that athletes, or those who regularly exercise, have a greater baseline bilirubin level of approximately 29 μM compared to non-athletes [10]. Unfortunately, there are not many studies that delve into the clinical outcome of elevated bilirubin during exercise.

It is possible that bilirubin protects muscle fibers from injury induced by exercise, delays the development of fatigue, and promotes recovery by inhibiting the disturbance of mitochondrial function [20,60]. While the effect of bilirubin on muscle tissue fibers is currently not known, we have previously shown that the UGT1A1*28 Gilbert’s polymorphism mice on a high-fat diet had greater lean body mass compared to high-fat-fed controls [23]. We also showed that treating obese mice with bilirubin nanoparticles increased lean body mass and mitochondrial function in white adipose tissue (WAT) [20]. Bilirubin may positively affect muscle tissue to increase lean body mass, or may also target adipose to reduce fat, or might benefit both to improve metabolic outcomes. Noland et al. showed that HCR rats had enhanced mitochondrial activity in muscle compared to the LCR [36]. Our results here indicate that the liver control of bilirubin may be a factor. However, both adipose or muscle tissues may be contributing to the mitochondrial function and higher plasma acetic and citric acid levels. Low intrinsic running capacity is associated with reduced skeletal muscle substrate oxidation and lower mitochondrial content in white skeletal muscle [61]. Adipose-specific BVRA knockout mice fed a high-fat diet exhibited increased fat mass and significantly fewer mitochondria in WAT than floxed control fed the same diet [38]. A liver to muscle or liver to adipose circuit may exist via BVRA mediated production of bilirubin, enhancing aerobic exercise capacity due to the mitochondrial effects of bilirubin [19,20,38,62], which might contribute to the high aerobic capacity of the HCR rats. Future studies should determine the metabolic actions of bilirubin and BVRA in WAT and adipose depots of the HCR and LCR.

We have recently shown that bilirubin has a hormonal function by binding directly to PPARα, inducing transcriptional activity of genes involved in hepatic glycogen synthesis and β-oxidation in models of dietary-induced non-alcoholic fatty liver disease (NAFLD) [18,19,20]. In the present study, we did not find a change in PPARα mRNA expression in the livers of the HCR compared to the LCR rats. This finding agrees with others who have also found that PPARα levels did not change with exercise in rats [28]. The interesting conundrum is that the PPARα transcriptional activity, but not expression, is higher during exercise [63,64]. The reason for the enhanced PPARα transcriptional activity could be due to PPARα ligands such as free fatty acids that are released from adipose tissue (lipolysis) [65] or increased bilirubin levels, both of which drive PPARα transcriptional activity. There has been a correlation for palmitoylethanolamide (PEA) and oleoylethanolamine (OEA), which are natural PPARα ligands derived from fatty acids, and their activation of PPARα may regulate bilirubin levels [66]. Others have proposed that PPARα may regulate bilirubin homeostasis [67]. These observations suggest a possible feedback loop for bilirubin-PPARα, which might be a fundamental circuit for exercise.

PPARα is known to increase glycogen content in the liver. Nolan et al. found no difference in glycogen synthesis in muscle tissue between the HCR and LCR rats [36]. However, we show here for the first time that glycogen was significantly higher in the livers of the HCR rats compared to the LCR. The increased glycogen stores in the livers of the HCR rats are most likely due to bilirubin-PPARα induction of glycogen synthase-2 (*Gys2*) [24]. There are hepatic pathways that are mediated by bilirubin that increases glycogen storage [15,68]. In muscle, the predominant isoform is *Gys1*, which is not known to be PPARα-regulated. It could be the reason for the lack of differences in muscle glycogen synthesis between HCR and LCR rats. The likely contributor to the higher hepatic glycogen content in the HCR rats is enhanced bilirubin-PPARα-GYS2. We have previously shown that mice with a hepatocyte-specific knockout of BVRA and reduced hepatic bilirubin had significantly lower glycogen content and suppressed PPARα activity [24]. We also demonstrated in the Gilbert’s (UGTA1A*28) mice with hyperbilirubinemia that they had significantly higher hepatic glycogen content on a high-fat diet compared to obese controls [23]. There may be a bilirubin-PPARα axis that is essential to regulate glycogen storage, and this is beneficial during exercise increasing aerobic capacity.

## 5. Conclusions

The present study demonstrates that rats genetically selected for high aerobic capacity exhibit increased plasma bilirubin levels due to alterations in key hepatic enzymes responsible for bilirubin generation or metabolism. These studies show that the mechanistic actions that regulate plasma bilirubin levels during exercise might be beneficial medically. Activating BVRA or inhibition of UGT1A1 may serve as therapeutics for improving exercise performance or weight management.
